# Unlocking Therapeutic Potential of Poly(Adenosine Diphosphate Ribose) Polymerase (PARP) Inhibitors in Metastatic Breast Cancer With BRCA Gene Mutations: A Narrative Review

**DOI:** 10.7759/cureus.46405

**Published:** 2023-10-03

**Authors:** Prateek Jain

**Affiliations:** 1 Internal Medicine, Maulana Azad Medical College, New Delhi, IND

**Keywords:** poly adenosine diphosphate-ribose polymerase inhibitors, metastatic triple-negative breast cancer, parp-inhibitors, brca gene mutation, : “breast cancer”

## Abstract

Breast cancer (BC), a significant global health concern, impacts millions of women worldwide. A key genetic factor in this disease is the presence of BReast CAncer gene (BRCA) mutations, which increase susceptibility to BC. This narrative review explores the crucial role of poly(adenosine diphosphate ribose) polymerase (PARP) inhibitors in treating metastatic BC in individuals with BRCA gene mutations. In BRCA mutation carriers, these inhibitors induce synthetic lethality, leading to cell death due to the accumulation of lethal DNA breaks. Clinical trials have demonstrated the effectiveness of PARP inhibitors, such as olaparib and talazoparib, in extending progression-free survival and response rates, especially in patients without prior chemotherapy. Moreover, this review discusses combination therapies, where PARP inhibitors are combined with cytostatic drugs like platinum-based chemotherapy. Some studies show the synergy of these approaches, even in patients without homologous recombination deficiency. In summary, PARP inhibitors offer hope for improving outcomes in metastatic BC patients with BRCA gene mutations. As research advances, PARP inhibitors continue to hold promise in the fight against BC.

## Introduction and background

Breast cancer (BC) is the second most prevalent cancer globally and is the leading malignancy among women, accounting for approximately 12% of all cancer diagnoses in 2018. BC-related deaths affected 627,000 women in the same year, making it the fourth leading cause of cancer-related mortality. While there is minimal male involvement (less than 1%), BC remains a significant public health concern. Risk factors encompass family history, age, environmental and lifestyle elements linked to carcinogen exposure, and hormonal fluctuations [1]. An estimated 5% of BC patients carry a germline BRCA mutation. These mutations are more prevalent in cases involving strong familial BC history, younger patients, those with triple-negative breast cancer (TNBC) indicating the absence of HER2, estrogen, and progesterone receptors, and specific ethnic groups, such as Ashkenazi Jews who exhibit BRCA gene founder mutations. BRCA1 mutation carriers are prone to TNBC, while BRCA2 mutation carriers often present estrogen receptor-positive tumors [2]. The risk of BC among BRCA1/2 mutation carriers is substantial, ranging between 50% and 85%. Within this group, approximately 70% are identified with the TNBC subtype, and among all TNBC patients, about 10-20% carry BRCA1/2 gene mutations [3]. BRCA1 and BRCA2 are vital in homologous recombination DNA repair. BCs featuring germline BRCA1 or BRCA2 pathogenic variants and biallelic inactivation demonstrate deficiencies in homologous recombination repair [4]. Cells harboring detrimental mutations in BRCA1/2 genes struggle to repair DNA double-strand breaks. This leads to heightened reliance on the single-strand break repair pathway, regulated by poly(adenosine diphosphate ribose) polymerase (PARP) inhibitors. For cells with BRCA1/2 mutations, PARP inhibition culminates in cell death due to the accumulation of irreparable DNA damage [5]. The PARP proteins, PARP1 and PARP2, are crucial in essential cellular functions like DNA repair and programmed cell death. Inhibiting PARP has emerged as an innovative strategy for cancer treatment, exploiting the DNA damage response weaknesses in cancer cells. With clinical trials underway, PARP inhibitors, like olaparib, veliparib, and iniparib, can potentially treat breast and ovarian cancers [6]. 

## Review

This review explores PARP inhibitor treatment for metastatic BC in patients with BRCA gene mutations, assessing their effectiveness, safety, and potential combination therapies. 

PARP and its action 

**Figure 1:**
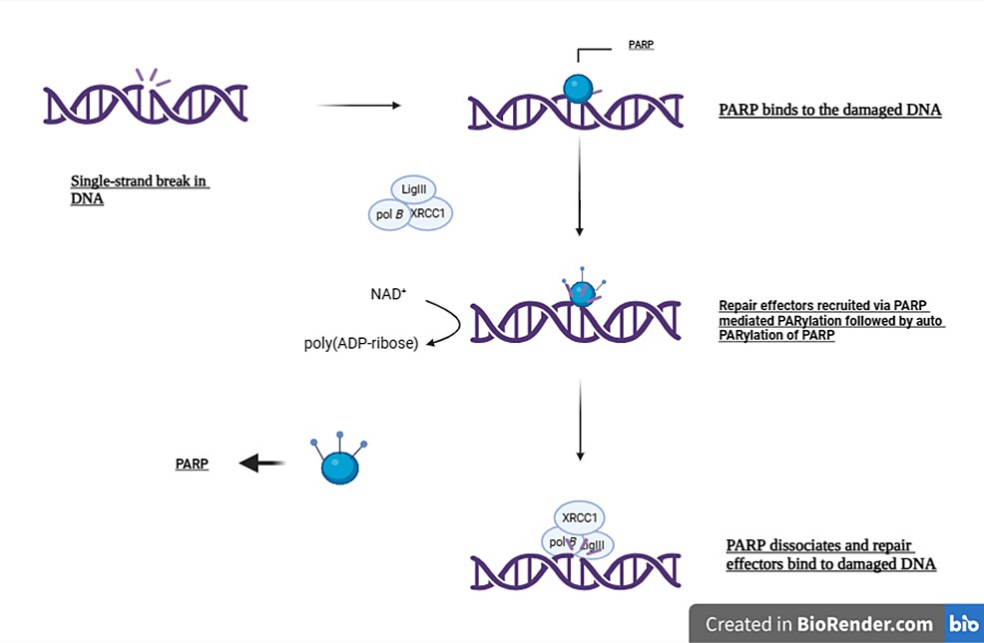
PARP's crucial role in repairing DNA single-strand breaks via the base excision repair process. LigIII: DNA ligase 3; NAD+: Nicotinamide Adenine Dinucleotide; PARP: Poly(ADP-ribose) polymerase; pol b: DNA Polymerase Beta; XRCC1: X-ray Repair Cross-Complementing Protein 1. Modified by Cortesi L et al. (2021) [1] under a Creative Commons Attribution-NonCommercial 4.0 International License.

PARP, a protein family characterized by structural resemblance and functional similarity, consists of enzymes such as PARP1 and PARP2. These proteins contain two ribose units and two phosphates per polymer unit. Specifically, PARP1 and PARP2 play a significant role in a DNA repair pathway called base excision repair (BER), addressing single-strand breaks (SSBs) [7]. Triggered by DNA damage, PARP activation modifies nuclear proteins, including histones, aiding DNA repair. PARP-1's early activation mainly helps create a DNA repair-friendly chromatin state by ADP-ribosylating histones and recruiting chromatin remodelers. Synthesized ADP-ribose by PARP acts as a "signal" for assembling DNA repair complexes at damage sites, mainly supporting BER and SSBs. PARPs' role in double-strand break repair, essential for error-free DNA repair, seems limited [8]. When PARP is suppressed, SSBs persist, leading to stalled replication forks and the eventual formation of double-strand breaks. PARP inhibitors disrupt the function, resulting in catalytic inhibition, and some of these inhibitors also trap PARP onto DNA. The resulting PARP-DNA complexes interfere with DNA replication. Recent investigations have highlighted the significance of PARP trapping in the cytotoxic effects of PARP inhibitors [9]. Figure 1 shows the role of PARP in the repair of SSBs in DNA [1].

PARP inhibitors and BRCA gene mutation 

BRCA1 and BRCA2 proteins are vital for repairing DNA breaks through homologous recombination repair (HRR). While BRCA1 detects and responds to DNA damage, BRCA2 governs key molecules like RAD51. RAD51, in conjunction with replication protein A (RPA), mends double-stranded breaks. This repair process involves both the accurate homologous recombination repair (HRR) and the swift, less precise non-homologous end joining (NHEJ) mechanisms. HRR utilizes the sister chromatid as a template, whereas NHEJ promptly seals DNA ends, potentially introducing sequence modifications. Together, these mechanisms ensure chromosome stability [10]. Tumors bearing BRCA mutations exhibit HRR deficiency due to a dual effect: a hereditary mutation in one allele and the inactivation of the other through loss of heterozygosity. When such tumors are administered with PARP inhibitors, it inevitably leads to the accumulation of DNA damage, promptly prompting cell-cycle arrest and programmed cell death. This response to PARP inhibition in cells with defective HRR flawlessly exemplifies synthetic lethality [9]. Synthetic lethality emerges when otherwise non-lethal conditions become lethal together. PARP inhibitors disrupt BER in cells with BRCA1 or BRCA2 mutations, causing cell damage. This is advantageous as it specifically targets tumor cells while sparing normal ones. As many BRCA mutation carriers have one abnormal allele and tumors acquire a second alteration, PARP inhibitors exploit synthetic lethality, killing tumor cells. This targeted approach avoids traditional treatment drawbacks like chemotherapy and radiation [7]. 

PARP inhibitors as monotherapy 

The US FDA and the European Medicines Agency (EMA) have approved the use of two PARP inhibitor monotherapies, olaparib and talazoparib, in patients with suspected or confirmed deleterious gBRCA mutations and HER2-negative BC. These approvals are based on positive outcomes from phase three trials, namely OlympiAD and EMBRACA [1, 11, 12]. The phase three OlympiAD trial aimed to assess the efficacy of olaparib versus standard chemotherapy (TPC) for treating HER2-negative metastatic BC patients who possessed a germline BRCA mutation. A total of 302 participants were enrolled and randomly divided into two groups: one receiving olaparib (205 patients) and the other TPC (97 patients). The study revealed that olaparib, taken orally as a monotherapy, significantly extended the median progression-free survival compared to TPC (7.0 months versus 4.2 months). The hazard ratio for the risk of disease progression or death was 0.58, indicating a reduced risk with olaparib. The 95% CI ranged from 0.43 to 0.80. These findings imply that olaparib could be a more effective treatment option for individuals with HER2-negative metastatic BC who have a germline BRCA mutation [2, 11]. In the EMBRACA trial, 431 patients were randomized, 287 receiving talazoparib and 144 standard therapy. Talazoparib significantly extended median progression-free survival (8.6 vs. 5.6 months) and had a higher objective response rate (62.6% vs. 27.2%). Talazoparib was associated with hematologic and nonhematologic adverse events, notably grade three to four hematologic problems (primarily anemia). Patient-reported outcomes favored talazoparib, significantly improving global health status and breast symptoms [5]. An analysis of two trials that pitted single-agent PARP inhibitor therapy against standard therapy in advanced BC patients with gBRCA1/2 mutations indicated that PARP inhibitor treatment (olaparib or talazoparib) correlated with enhanced complete response rates and increased progression-free survival rates [13]. Numerous phase one and two studies have highlighted the single-agent efficacy of PARP inhibitors in metastatic BC patients with germline BRCA mutations [13, 14]. Among Asian patients in the Olaparib arm, a more prolonged median progression-free survival was observed compared to those receiving chemotherapy TPC (5.7 vs. 4.2 months), consistent with the global findings. This finding was supported by both blinded independent central review (BICR)-assessed and investigator-assessed progression-free survival [11, 14]. In summary, the use of PARP inhibitors such as olaparib and talazoparib in the OlympiAD and EMBRACA trials demonstrated improved progression-free survival and response rates compared to standard therapy for HER2-negative metastatic BC with gBRCA mutations, particularly in patients with no prior chemotherapy. These results suggest the potential benefit of incorporating PARP inhibitors earlier in the treatment course. However, optimizing BRCA testing and establishing the appropriate selection and sequencing of PARP inhibitors is crucial for enhancing outcomes and guiding clinical decisions in this patient population [11, 12, 15]. 

The safety profile of PARP inhibitors 

Olaparib displayed a safety profile that was both well-tolerated and manageable, aligning with previous research findings. Severe Treatment-Emergent Adverse Events (TEAEs) were relatively minimal at 25.4%, a figure comparable to the 36.6% observed in the olaparib arm of the OlympiAD study. The median treatment duration in the study was 7.9 months, closely mirroring the 8.2 months noted in OlympiAD. Moreover, TEAE-related discontinuations were uncommon in both the LUCY phase IIIb trial (4.4%) and OlympiAD (4.9%). No new safety concerns were identified, and there were no instances of myelodysplastic syndrome (MDS) or acute myeloid leukemia (AML) [11,16]. Olaparib showcased benefits over standard therapy, reflecting fewer adverse events of grade three or higher and fewer discontinuations due to adverse events. The olaparib group commonly experienced nausea of grade one or two, while anemia was the predominant event of grade three or higher. The safety profile of olaparib remains consistent with earlier studies [2]. Around 55% of talazoparib-treated patients had grade three or four hematologic adverse events, higher than the 38% in chemotherapy-treated patients. PARP inhibitor monotherapy-related adverse events involve gastrointestinal symptoms, hematological effects, and fatigue. Certain adverse events are more familiar with specific inhibitors, such as olaparib linked to hepatotoxicity and niraparib to thrombocytopenia. "Off-target" profiles of PARP inhibitors lead to diverse effects. A minimal (<1%) long-term risk of MDS and AML was seen in clinical trials [17]. In a meta-analysis evaluating the efficacy and safety of PARP inhibitors, utilized either concomitantly or independently of chemotherapy, compared to chemotherapy alone for advanced BC, a noteworthy observation was that severe adverse effects (grade ≥3) were more prevalent in regimens incorporating PARP inhibitors than in chemotherapy-alone protocols. Specifically, the incidence of thrombocytopenia was significantly higher in the PARP inhibitor-containing regimens. Although there was no substantial difference in the occurrence of severe anemia, there was a tendency towards a higher rate in the group treated with PARP inhibitors. Notably, there were no significant differences in the incidence of severe fatigue between the two groups [18]. Talazoparib's safety profile was consistent with earlier reported data. Most grade three to four adverse events were hematologic and managed with supportive care, including transfusions and dose adjustments. A 5.9% rate of permanent treatment discontinuation due to adverse events was observed. In the EMBRACA trial, anemia and fatigue were more frequent in talazoparib-treated patients (13.6%) than in chemotherapy recipients (4.0%). A few cases of grade three fatigue were reported (2.4% talazoparib vs. 3.2% chemotherapy) [12]. 

Combination therapies 

As previously mentioned, while PARP inhibitor monotherapy has shown favorable patient outcomes, preliminary studies suggest that combining PARP inhibitors with cytostatic drugs before or after treatment may yield even more promising results. Indeed, the treatment of TNBC patients with PARP inhibitors in combination with platinum-based chemotherapy, either before or after, has demonstrated beneficial outcomes in the OlympiAD and EMBRACA open-label randomized phase three trials, utilizing olaparib and talazoparib, respectively. This finding is particularly significant given that approximately two-thirds of triple-negative patients possess proficient BRCA1 and lack homologous recombination deficiency [10,11,12]. Extensive research into combining PARP inhibitors for metastatic BC, particularly veliparib, reveals promising synergy with cytotoxic agents, enhancing effects even in tumors that are unresponsive. Clinical trials, including a phase two study for BRCA-associated BCs, demonstrate a 22% response rate and a 50% clinical benefit rate when paired with temozolomide. These results are corroborated in patients who have been previously treated with platinum compounds or PARP inhibitors [8]. PARP inhibition heightens tumor cell sensitivity to DNA-damaging treatments like platinum agents, temozolomide, and radiation. Clinical trials explore strategies combining these therapies to boost cytotoxic chemotherapy effects. Notably, veliparib is a focus of these studies. A dose escalation trial combining veliparib, carboplatin, and paclitaxel in advanced solid tumor patients, particularly BC, showed promising activity, with a 57% objective response rate [9]. A promising phase one trial combining cisplatin, vinorelbine, and veliparib demonstrated both tolerability and efficacy for advanced TNBC and BRCA-mutated BC patients. Veliparib's recommended phase II dose was established at 300 mg twice daily, aligning closely with its maximal monotherapy dose and showcasing clinical activity as a single agent. Notably, the anti-neoplastic activity was observed in patients regardless of the presence of germline BRCA mutations [19]. A phase I study using a dose-escalation approach aimed to find the recommended phase two dose (RP2D) for combining oral veliparib and pegylated liposomal doxorubicin (PLD) in metastatic TNBC or recurrent ovarian cancer patients. Forty-four participants were examined across different doses of veliparib (50 mg to 350 mg) and PLD (22.5-40 mg/m2). The RP2D established was veliparib 200 mg twice daily on days 1-14 alongside PLD at 40 mg/m2 on day one within a 28-day cycle [20]. 

## Conclusions

In summary, PARP inhibitors like olaparib and talazoparib show promise as standalone treatments for gBRCA-mutated HER2-negative breast cancer, notably improving progression-free survival and response rates. These therapies exhibit generally manageable side effects with low treatment discontinuation rates. Although rare, vigilance for long-term risks like MDL and AML is necessary. Combining PARP inhibitors with cytostatic drugs, such as platinum-based chemotherapy, has benefits, even in TNBC patients without homologous recombination deficiency. Preclinical data supports the synergy between PARP inhibitors and cytotoxic compounds, offering potential for treatment optimization. Integrating PARP inhibitors into combination regimens may enhance therapeutic responses and guide clinical decisions, requiring ongoing refinement in patient selection, treatment sequencing, and monitoring strategies.
